# Comparing the Cervista HPV HR Test and Hybrid Capture 2 Assay in a Dutch Screening Population: Improved Specificity of the Cervista HPV HR Test by Changing the Cut-Off

**DOI:** 10.1371/journal.pone.0101930

**Published:** 2014-07-22

**Authors:** Aniek Boers, Lorian Slagter-Menkema, Bettien M. van Hemel, Jerome L. Belinson, Teus Ruitenbeek, Henk J. Buikema, Harry Klip, Hilde Ghyssaert, Ate G. J. van der Zee, Geertruida H. de Bock, G. Bea A. Wisman, Ed Schuuring

**Affiliations:** 1 Department of Obstetrics and Gynecology, Division of Gynecologic Oncology, University of Groningen, University Medical Center Groningen, Groningen, the Netherlands; 2 Department of Pathology and Medical Biology, University of Groningen, University Medical Center Groningen, Groningen, the Netherlands; 3 Preventive Oncology International, Inc, Cleveland Heights and Lerner College of Medicine, Cleveland Clinic, Cleveland, Ohio, United States of America; 4 Department of Pathology, AZ St Jan Brugge-Oostende, Brugge, Belgium; 5 Department of Epidemiology, University of Groningen, University Medical Center Groningen, Groningen, the Netherlands; Fondazione IRCCS Policlinico San Matteo, Italy

## Abstract

The diagnostic performance of the widely-used Cervista HPV HR test was compared to the Hybrid Capture 2 (HC2) test in a Dutch population-based cervical cancer screening program. In 900 scrapings of women with normal cytomorphology, specificity was 90% (95%CI: 87.84–91.87) for the Cervista HPV HR test and 96% (95%CI: 94.76–97.37) for the HC2 test with 93% agreement between both tests (κ = 0.5, p<0.001). The sensitivity for CIN2+ using 65 scrapings of women with histological-confirmed CIN2+ was 91% (95%CI: 80.97–96.51) for the Cervista HPV HR test and 92% (95%CI: 82.94–97.43) for the HC2 test with 95% agreement between both tests (κ = 0.7, p<0.001). Fifty-seven of 60 HC2 negative/Cervista positive cases tested HPV-negative with PCR-based HPV assays; of these cases 56% were defined as Cervista triple-positive with FOZ values in all 3 mixes higher than the second cut-off of 1.93 (as set by manufacturer). By setting this cut-off at 5.0, specificity improved significantly without affecting sensitivity. External validation of this new cut-off at 5.0 in triple-positive scrapings of women selected from the SHENCCASTII database revealed that 22/24 histological normal cases now tested HPV-negative in the Cervista HPV HR test, while CIN2+ lesions remained HPV-positive. The intra-laboratory reproducibility of the Cervista HPV HR test (n = 510) showed a concordance of 92% and 93% for cut-off 1.93 and 5.0 (κ = 0.83 and κ = 0.84, p<0.001) and inter-laboratory agreement of the Cervista HPV HR test was 90% and 93% for cut-off 1.93 and 5.0 (κ = 0.80 and κ = 0.85, p<0.001). In conclusion, the specificity of the Cervista HPV HR test could be improved significantly by increasing the second cut-off from 1.93 to 5.0, without affecting the sensitivity of the test in a population-based screening setting.

## Introduction

Population-based screening programs have led to a significant reduction of the incidence and mortality from cervical cancer [1]. In the Netherlands cytomorphological examination of cervical scrapings is used for early detection of cervical cancer and premalignant cervical intraepithelial neoplasia (CIN). Despite the high specificity (95–97%), a disadvantage of cytomorphological examination is the relatively low sensitivity (50–60%) for detection of high grade CIN lesions (CIN2/3) and cervical cancer [2].

Cervical carcinogenesis is strongly associated with high-risk human papillomavirus (hrHPV). Persistent infection with hrHPV can result in CIN lesions and neoplastic progression. Testing for hrHPV in cervical scrapings shows high sensitivity (94–97%) to detect CIN2+ lesions. However specificity, especially in younger women, is around 6% lower than with cytology [2], [3]. Nowadays cervical cancer screening programs in many countries have combined cytomorphological examination and hrHPV testing [4], [5]. The current Dutch screening program is primarily based on cytomorphological classification with hrHPV testing as a triage test for abnormal cytological results (ASCUS/LSIL) [4]. In the Netherlands the population-based screening program will change to primary hrHPV screening in 2016 [6]. In primary screening hrHPV testing will be performed mostly on scrapings with no abnormalities, since the majority of the screening population is healthy. An optimal balance between the sensitivity and specificity of the hrHPV test is therefore important. At this moment numerous hrHPV tests are available, but only seven tests have been approved by the United States Food and Drug Administration (FDA) [7]–[9].

The first 2 and mostly used FDA approved HPV tests are the Hybrid Capture 2 (HC2) and the Cervista HPV HR assay [10]. The Digene HC2 test (Qiagen, Gaithersburg, MD) is a nucleic acid hybridization assay with signal amplification using microplate chemiluminescence for the detection of HPV DNA from 13 hrHPV types [11], [12]. The Cervista HPV HR test (Hologic Inc., Madison, WI, USA) uses Invader chemistry, a signal amplification method for detection of specific nucleic acid sequences [13], [14]. The Cervista HPV HR test detects 14 hrHPV types: HPV66 and the same 13 hrHPV types as detected by the HC2 test. Advantages of the Cervista HPV HR test compared to the HC2 test are; reduced sample volume required for testing (2 ml vs. 4 ml), the presence of an internal control which reduces the possibility of false-negative results due to insufficient DNA present in the sample and significant lower cross-reactivity to other HPV types [13], [15], [16].

Several studies analyzed the sensitivity and specificity for either the Cervista HPV HR test or the HC2 test [2], [13], [15], [17]–[20], but studies comparing both assays on the same samples in a population-based screening setting are limited [21]–[23]. In this study, we compared the performance of the widely-used Cervista HPV HR test with the “golden standard” HC2 test on the same scrapings selected from the national population-based cervical cancer screening based on the international guidelines for HPV DNA testing in primary cervical cancer screening in women 30 years and older [24]. Samples with discordant results were analyzed using additional PCR-based HPV detecting assays. In addition, we determined the intra-laboratory reproducibility and inter-laboratory agreement of the Cervista HPV HR test.

## Materials and Methods

### Sample collection

To compare the specificity of the Cervista HPV HR and HC2 test, 900 cytomorphological normal cervical scrapings (NILM) collected in PreservCyt of women between the ages of 30–60 years were randomly selected from the routine Dutch population-based screening program. Since women without cytomorphological abnormalities are not referred to the hospital for colposcopy, histology is not available for this group. To compare the specificity we only included women who also had a normal cervical scraping at the previous population-based screening 5 years prior and are therefore with the smallest chance of having an undetected CIN2+ lesion. Women with a history of (pre)malignant cervical lesions, abnormal cervical smears or any surgery in the area of the cervix as well as HIV-seropositive or pregnant women were excluded. Study-specific, uniquely numbered samples with more than 12 ml residual PreservCyt solution were collected to perform Cervista HPV HR and HC2 testing.

To compare the sensitivity of the Cervista HPV HR and HC2 test, we randomly selected scrapings of women referred to the University Medical Center with abnormal cytology (>BMD) during routine population-based screening. All 65 women included had histological confirmed CIN2+ lesions. Since a considerable number of CIN2+ lesions are missed by routine cytomorphological examination [2], we also included, of these 65 patients, 17 patients with a normal cytomorphological diagnosis [25]. These samples were selected from our research database of women who underwent a new cervical scraping before colposcopy.

### Cervista HPV HR method

The Cervista HPV HR test (Hologic Inc., Madison, WI, USA) is a qualitative test detecting 14 hrHPV types (HPV 16, 18, 31, 33, 35, 39, 45, 51, 52, 56, 58, 59, 66 and 68) [13], [14]. The assay uses three separate oligonucleotide mixtures; Mix 1 (A5/A6 pool) contains probes for HPV 51, 56 and 66; mix 2 (A7 pool) probes for HPV 18, 39, 45, 59 and 68, and mix 3 (A9 pool) probes for HPV 16, 31, 33, 35, 52 and 58. In these three mixes, oligonucleotides for the human histone 2 gene (HIST2H2BE) are also present as an internal control for the presence of sufficient genomic DNA [14]. A signal to noise value (sample signal measured against signal from a No Target Control) is generated for each of the three mixes and is referred to as HPV Fold-Over-Zero (FOZ). The HPV FOZ ratio is calculated by dividing the highest FOZ value from any one of the three reaction mixtures by the lowest HPV FOZ value of the three mixtures. If the HPV FOZ ratio is equal to or greater than 1.525, the sample is considered positive for hrHPV [14]. Samples with mixed HPV infections might result in positive signals of similar intensity in two or three reaction wells. Therefore, if the HPV FOZ ratio is lower than 1.525, but the HPV FOZ values in all three mixes are larger than the second cut-off value at 1.93 (default setting), the sample is considered positive for hrHPV in the Cervista HPV HR test [14].

### HC2 method

The HC2 test is routinely used in our (ISO15189 certified) laboratory. The HC2 test is clinically validated and FDA-approved and detects 13 hrHPV types (HPV 16, 18, 31, 33, 35, 39, 45, 51, 52, 56, 58, 59 and 68). The HC2 test has previously been described extensively and results are interpreted as a ratio of relative light units (RLU/CO) to the positive control specimen [11], [12]. Samples with an RLU/CO ratio >1.0 are considered positive for hrHPV. If the RLU/CO ratio <1 the sample is negative for hrHPV infection and borderline RLU/CO ratios (1–2.5) are re-tested.

### GP5+/6+ PCR and INNO-LiPA genotyping assay

All 965 specimens were tested both with the Cervista HPV HR test and HC2 test. Cases with discordant results were retested for the presence of hrHPV using PCR-based HPV detection assays. The HPV-L1 consensus GP5+/6+ PCR was performed as previously described [26] on DNA extracted for the Cervista HPV HR test. Samples positive for the GP5+/6+ HPV-PCR were defined as true HPV-positive cases. The genotype of L1-HPV PCR positive cases was determined utilizing the INNO-LiPA HPV genotyping *Extra* assay [27], [28]. For quality control, genomic DNA was amplified in a multiplex PCR containing a control gene primer set resulting in products of 100, 200, 300, 400 and 600 bp according to the BIOMED-2 protocol [29]. Only DNA samples with PCR products of 300 bp and larger were used for the detection of HPV.

### In silico analysis of the SHENCCASTII data

To evaluate the effect of different second threshold values for the Cervista HPV HR test we used an external patient group with histological-confirmed normal and abnormal tissue. In silico analysis of the data available from the Shenzhen Cervical Cancer Screening Trial II (SHENCCASTII) [21] was kindly provided by dr. S. Belinson. From the SHENCCASTII dataset a cohort comparable to our dataset was composed. This cohort contained data of women between the age 30–60 years who had a cervical scraping obtained by a physician (self-sampling scrapings were excluded) and HC2 as well as Cervista HPV HR results. All hrHPV positive women were referred for colposcopy and histological diagnosis was available.

### Intra- and inter-laboratory reproducibility of the Cervista HPV HR test

For intra- and inter-laboratory variability of the Cervista HPV HR test, 510 scrapings were selected. Seventy samples were selected from the 900 cytomorphological normal women from the population-based screening program. In the Netherlands women diagnosed with ASCUS or low-grade SIL are retested 6 months later using both cytomorphological assessment as well as hrHPV testing according the Dutch guidelines [4]. From these triage samples, 186 hrHPV-HC2 positive and 254 hrHPV-HC2 negative randomly-selected scrapings were included in this study according to the international guidelines for HPV DNA testing in primary cervical cancer screening in women 30 years an older by Meijer et al [24]. To determine the intra-laboratory reproducibility, all 510 samples were tested twice at different time points (at least 1 week difference) by the same experienced technician. For the inter-laboratory agreement, 2 ml PreservCyt of the same samples were send to an independent reference-laboratory using Cervista HPV HR testing routinely (Department of Pathology, AZ St Jan Brugge-Oostende, Brugge, Belgium). All samples were randomly-renumbered and provided to the reference-lab without knowledge of any results from the UMCG on cytomorphology or HPV status.

### Patient data

Clinicopathological data of the patients such as age, medical history, cytological and histological results were retrieved from the hospital database and the patient's pathology report, and entered into a separate, anonymous, password protected database. Protection of patient identity was guaranteed by assigning study-specific unique patient numbers ensuring that data is not traceable to individual patients. Codes were only known to one data manager. Therefore, according to the Dutch Law for Human Medical Research (WMO), no consent was necessary from the medical ethics committee for this study.

### Statistical analysis

Statistical analysis was performed using SPSS software, version 18 (SPSS Inc., Chicago, IL). The number of cases needed for the comparison of the specificity, sensitivity, inter- and intra laboratory variability were calculated from the power analysis described by the international guidelines for HPV DNA tests for primary cervical screening in women 30 years and older by Meijer et al [24]. To calculate the specificity of the Cervista HPV HR test and the HC2 test, the number of true negatives (HPV negative and cytomorphologically normal) was divided by the total number of healthy individuals (n = 900). The sensitivity was calculated by dividing the number of true positives (HPV positive and with CIN2+ lesion) with the total number of patients with CIN2+ lesions (n = 65). Agreement between both tests was calculated by Cohen's kappa. Triple positive cases in the study were combined to determine the best HPV FOZ second cut-off value for discriminating between true-negative and true-positive HPV cases. The statistical analysis on the SHENCCASTII data set was performed at the Preventive Oncology International Center for Biostatistics and Epidemiology (Cleveland Heights, Ohio) kindly provided by dr. S. Belinson.

## Results

### Sensitivity and specificity results in a Dutch screening population

In scrapings of 65 women with histological confirmed CIN2+ lesions, sensitivity of the Cervista HPV HR test was 91% (95%CI: 80.97–96.51), for the HC2 test this was 92% (95%CI: 82.94–97.43) (Table 1 and Table 2). Comparing both assays revealed a 95% agreement with a kappa of 0.7 (p<0.001).

**Table 1:** Performance of the Cervista HPV HR test in women aged 30 years and older.

	Women with CIN2+	Women without ≥CIN2+	Total
**Cervista HPV HR test positive**	59	90	149
**Cervista HPV HR test negative**	6	809	815
**Low gDNA**	0	1	1
**Total**	65	900	965

**Table 2:** Performance of the Hybrid Capture 2 assay in women aged 30 years and older.

	Women with CIN2+	Women without ≥CIN2+	Total
**HC2 test positive**	60	34	94
**HC2 test negative**	5	866	871
**Total**	65	900	965

The specificity of the Cervista HPV HR and the HC2 test in 900 cytomorphological normal cervical scrapings was 90% (95%CI: 87.84–91.87) and 96% (95%CI: 94.76–97.37), respectively (Table 1 and Table 2). Comparison revealed an agreement of 93% between both tests with a kappa of 0.47 (p<0.001). The prevalence rate for detecting HPV in the cytomorphological negative population was 10% (90/899) using Cervista HPV HR test and 4% (34/900) using the HC2 test.

### Characterization of discordant results between the Cervista HPV HR and HC2 test

Of the total 965 cases, 66 cases showed discordant results when comparing the Cervista HPV HR and HC2 test. One HC2-negative case, showed a low gDNA outcome in the Cervista HPV HR test. Re-testing of this sample with the Cervista HPV HR test again showed a low gDNA outcome. This could be a false-negative result in the HC2 test, because of insufficient cells in the sample. Cytological examination confirmed low number of cells in the sample.

Five cases were HC2 positive and Cervista negative (Table 3). Using the PCR-based consensus L1-HPV test (GP5+/6+ PCR) 4 out of 5 were positive. HPV typing according to the INNO-LiPA assay (Table 3 and Table S1) showed multiple HPV types in the tested samples.

**Table 3:** Discordant HC2 positive/Cervista HPV HR negative samples.

*Nr*	*HC2 result*	*Cervista result*	*Cervista re-test*	*GP5+/6+ L1-PCR*	*INNO-LiPA HPV genotyping*
2	Positive	Negative	Negative	Negative	HPV33
3	Positive	Negative	Negative	Positive	HPV51
4	Positive	Negative	NP	Positive	NP*
5	Positive	Negative	NP	Positive	HPV33, 69, 71
6	Positive	Negative	Positive (mix 1)	Positive	HPV53, 54, 66

DNA from the initial Cervista HPV HR test was used for re-testing with the Cervista HPV HR test, the GP5+/6+PCR and for HPV-typing using INNO-LiPA in the GP5+/6+ positive cases. For some tests insufficient material was available.

*NP =  not performed because of insufficient material.

Most discordant cases (n = 60) reported HC2 negative/Cervista positive cases. The GP5+/6+ PCR was performed to determine if hrHPV DNA was in fact present in each of these samples (Table S1). Three cases were positive and genotyping with the INNO-LiPA assay revealed HPV 39 and 56 (nr 34), HPV 16 (nr 35) and HPV 44 and 56 (nr 61). Thus the HC2 assay gave false-negative results in 3 of the 60 (5%) discordant cases tested. Remarkably, all other discordant cases tested negative with the GP5+/6+ PCR, resulting in false-positive results for the Cervista HPV HR test in 57 of the 60 cases (95%). Of these 57 HC2 negative/Cervista positive cases, 18 samples were positive in mix 1, 5 samples in mix 2, 2 samples in mix 3 and 32 samples in all 3 mixes (so-called Cervista triple-positive cases). Re-testing of these 57 discordant cases with the Cervista HPV HR test revealed 24 negative and 32 HPV positive cases (Table S1).

### Improving specificity of the Cervista HPV HR test by increasing the second cut-off value

In the Cervista HPV HR test, cases with a HPV FOZ ratio <1.525 are considered HPV-negative except those cases where all three mixes have a HPV FOZ >1.93, referred to as triple-positive cases [13], [14]. In the group of 57 discordant HC2-negative/Cervista HPV HR positive cases, 32 (56%) cases were Cervista triple-positive (Table S1 and Table S2). These cases were obtained from cytomorphologically negative women and tested negative using the GP5+/6+ PCR and are therefore defined as true-HPV-negative cases.

We noticed that the lowest HPV FOZ mix value in the Cervista triple-positive cases varied between 1.95 and 4.60; only one case showed higher HPV FOZ mix values (6.58/6.83/6.22) (Table S2). Since these 32 triple-positive samples were part of our series of 900 cytomorphological normal cervical scrapings, thereby representing a group with the smallest chance of having an undetected CIN2+ lesion, increasing the second HPV FOZ cut-off value of 1.93 might improve the specificity of the Cervista HPV HR test.

**Figure 1:**
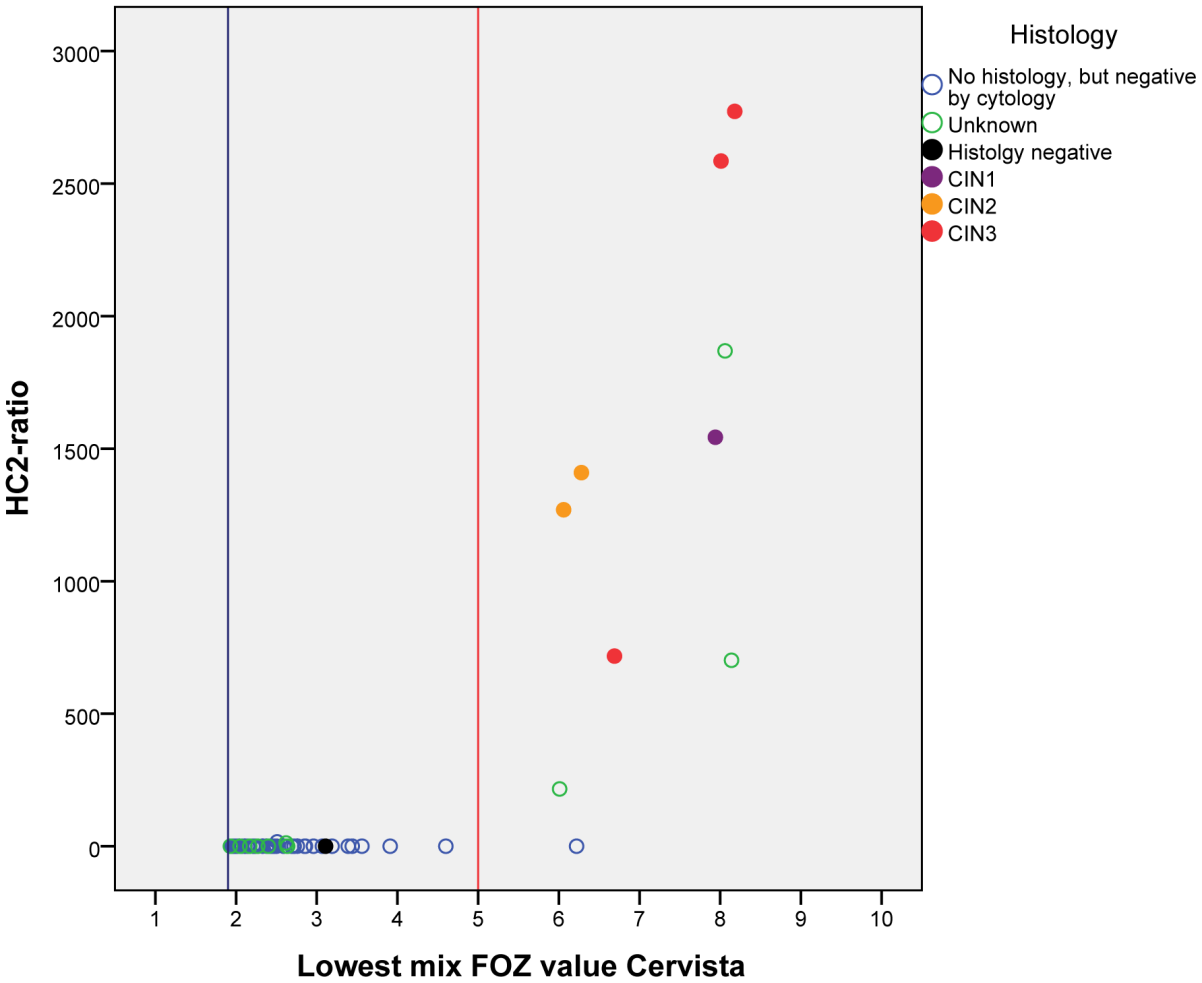
The lowest mix HPV FOZ value of the Cervista HPV HR test versus the HC2 ratio value in the 63 Cervista triple-positive cases. The blue line marks the default second cut-off at 1.93 of the Cervista HPV HR test; the red line marks the cut-off set at 5.0.

To determine the best HPV FOZ second cut-off value for discriminating between true-negative and true-positive HPV cases, we included all observed triple positive cases in this study. In addition to the 32 discordant triple-positive cases from the cytomorphological normal scrapings, in our whole cohort of 1405 samples (including samples used for intra- and inter-laboratory testing), we observed 31 additional Cervista triple-positive cases including scrapings with abnormal cytomorphology and/or HPV-positivity (Table S3). In this group the lowest HPV FOZ mix value varied between 1.93 and 8.18. Of these 31 cases, 11 were HC2 positive. Comparing the lowest FOZ mix value of the three mixes in the Cervista HPV HR test with the HC2 ratio of all 63 Cervista triple-positive cases revealed that the second cut-off of 1.93 (default setting) is not optimal (see blue vertical line in Figure 1). Increasing the cut-off to 5.0, all but one (nr 12) of the 52 HC2-negative cases are now correctly classified as Cervista HPV-negative, whereas only 2 HC2-positive (nr 40 en 41) are now considered as Cervista-negative. All histological confirmed CIN2+ lesions remained positive.

To evaluate the effect of different second cut-off values, we re-calculated sensitivity and specificity of the Cervista HPV HR test on our series of 900 women with cytomorphological negative scrapings and on the 65 scrapings associated with histological proven CIN2+ lesions (Table 4). Increasing the second cut-off to 5.0 improved the specificity of the Cervista HPV HR test in a cytomorphological normal population from 90.0% to 93.4%. Sensitivity of the test was not affected when increasing the second cut-off to 5.0 (Table 4). Comparing the specificity of the Cervista HPV HR test (using this new cut-off of 5.0) with the HC2 test in our group of 900 cytomorphological normal scrapings, agreement between both tests improved from 93% to 97% (kappa improved from 0.47 to 0.67) (p<0.001).

**Table 4:** Sensitivity and specificity of the Cervista HPV HR test using different second HPV FOZ cut-off values.

Second cut-off	Specificity Cervista	Sensitivity Cervista
1.93	809/899 = 90.0%	59/65 = 90.8%
3.0	833/899 = 92.7%	59/65 = 90.8%
4.0	839/899 = 93.3%	59/65 = 90.8%
**5.0**	**840/899 = 93.4%**	**59/65 = 90.8%**
6.0	840/899 = 93.4%	58/65 = 89.2%
7.0	841/899 = 93.5%	58/65 = 89.2%

However, improving sensitivity and specificity of the Cervista HPV HR test is not solely dependent on the HPV status of the scraping, but primarily by the presence of histological confirmed CIN2+ lesions. By law in most countries, including the Netherlands, no colposcopy is performed on women with normal cytomorphology. Consequently, in our series of 63 Cervista triple-positive cases only from 6 women histology was available. In five cases CIN2 or CIN3 lesions were detected and all showed a second cut-off above 5.0 (Figure 1 and Table S3). From the Cervista triple-positive cases with normal cytomorphology 44 out of 45 scrapings had a second cut-off below 5.0 (Table S2). Only 1 HC2-negative case with normal cytology (nr 12) showed a second cut-off above 5.0.

**Figure 2:**
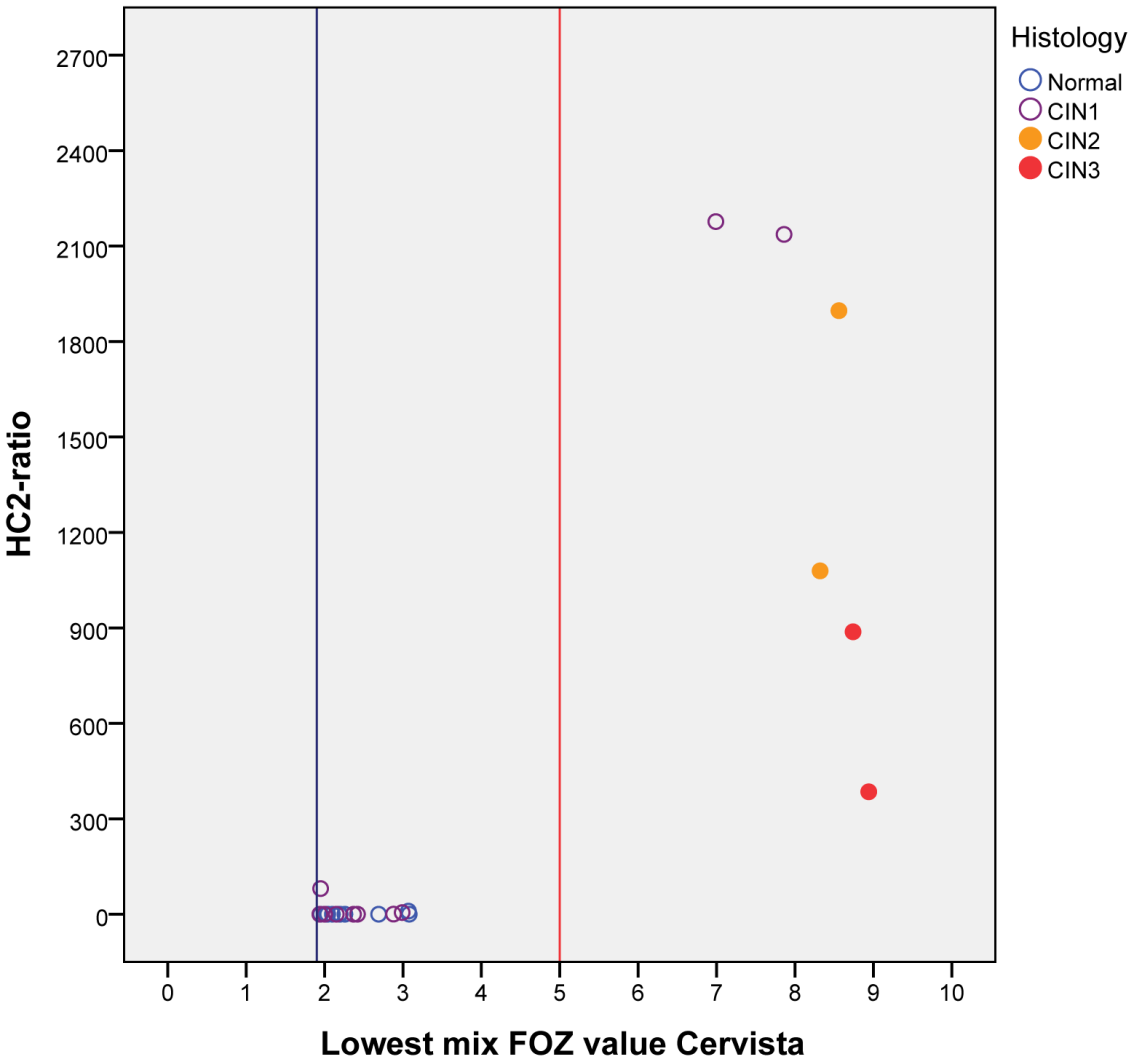
Comparison of the lowest mix HPV FOZ value of the Cervista triple-positive cases with HC2 ratio and the corresponding histological diagnosis of the biopsy. The 28 Cervista triple-positive cases with histological available were retrieved from the SHENCCASTII dataset.

To evaluate the effect of a different second cut-off for the Cervista HPV HR test on patients with histological diagnosis, we analyzed in silico an independent external cohort from the SHENCCASTII dataset. In the SHENCCASTII study women were referred for colposcopy if they were positive on any of the HPV tests performed. In addition, every HPV positive woman referred to colposcopy had a minimum of 5 cervical biopsies [21]. This means that women with cytomorphological normal scrapings but positive for hrHPV were subjected to colposcopy and histological examination. From this cohort, 28 Cervista triple-positive cases with histological diagnosis were retrieved (Table S4). All 6 cases with a high HC2 ratio (>380) showed a lowest FOZ mix value above the new second cut-off of 5.0 including 4 cases with CIN2 or CIN3 (Figure 2). Also, 3 cases with relative low HC2 ratio as well as all 19 HC2-negative cases showed a lowest FOZ mix value below the second cut-off value of 5.0 (Figure 2). These 22 scrapings would be considered as HPV-negative using the new second cut-off at 5.0 and are all associated with normal (≤CIN1) histological results (Figure 2).

### The intra- and inter-laboratory reproducibility of the Cervista HPV HR test

To ensure a reliable performance of the Cervista HPV HR test in clinical practice, we validated the intra-laboratory reproducibility and inter-laboratory agreement in time. The intra laboratory reproducibility (n = 510) showed a concordance of 92% and 93% with a kappa of 0.83 and 0.84 for cut-off 1.93 and 5.0 respectively (p<0.001) (Table 5). The inter-laboratory agreement between our laboratory and an independent laboratory that uses the Cervista HPV HR test routinely on the same 510 scrapings showed agreement between the two laboratories of 90% and 93% with a kappa of 0.80 and 0.85 for cut-off 1.93 and 5.0 (p<0.001) (Table 6).

**Table 5:** Intra-laboratory reproducibility of the Cervista HPV HR test with a second cut-off at default setting of 1.93 (A) and at new setting of 5.0 (B).

*(A) Cut-off 1.93* *	*Cervista test 2 positive*	*Cervista test 2 negative*	*Low gDNA*	*Total*
***Cervista test 1 positive***	174	24	0	**198**
***Cervista test 1 negative***	17	293	1	**311**
**Low gDNA**	0	0	1	**1**
**Total**	**191**	**317**	**2**	**510**

*(B) Cut-off 5.0* **	*Cervista test 2 positive*	*Cervista test 2 negative*	*Low gDNA*	*Total*
***Cervista test 1 positive***	169	21	0	**190**
***Cervista test 1 negative***	16	302	1	**319**
**Low gDNA**	0	0	1	**1**
**Total**	**185**	**323**	**2**	**510**

The same sample was tested twice by the same technician within an interval of 1–3 weeks.

*Concordance of the 510 scrapings tested twice was 92% (kappa of 0.83; p<0.001).

**Concordance of the 510 scrapings tested twice was 93% (kappa of 0.84; p<0.001).

**Table 6:** Inter-laboratory agreement of the Cervista HPV HR test with a second cut-off at default setting of 1.93 (A) and at new setting of 5.0 (B).

*(A) Cut-off 1.93* *	*Cervista test Brugge positive*	*Cervista test Brugge negative*	*Low gDNA*	*Total*
***Cervista test UMCG positive***	179	12	0	**191**
***Cervista test UMCG negative***	35	281	1	**317**
**Low gDNA**	0	1	1	**2**
**Total**	**214**	**294**	**2**	**510**

*(B) Cut-off 5.0* **	*Cervista test Brugge positive*	*Cervista test Brugge negative*	*Low gDNA*	*Total*
***Cervista test UMCG positive***	175	10	0	**185**
***Cervista test UMCG negative***	25	298	0	**323**
**Low gDNA**	1	1	0	**2**
**Total**	**201**	**309**	**0**	**510**

Two ml PreservCyt of samples tested in our laboratory (UMCG) were sent to another laboratory (Brugge in Belgium) that uses the Cervista HPV HR assay routinely.

*Concordance between 2 laboratories (UMCG-Groningen and Ghent) on the same 510 scrapings was 90% (kappa of 0.80; p<0.001).

**Concordance between 2 laboratories (UMCG-Groningen and Ghent) on the same 510 scrapings was 93% (kappa of 0.85; p<0.001).

## Discussion

The aim of the present study was to compare the diagnostic performance of the Cervista HPV HR test versus the HC2 test on the same cervical scrapings from women participating in the routine Dutch population-based screening program. The sensitivity for detecting CIN2+ lesions in a cohort of women referred with an abnormal scraping was comparable between the Cervista HPV HR test (91%) and the HC2 assay (92%). The specificity in a cohort of 900 women with repeated normal cytomorphology was 96% for the HC2 test versus 90% in the Cervista HPV HR test. However, by adjusting the second threshold to 5.0 we were able to improve the specificity of the Cervista HPV HR test to 93% without affecting the sensitivity. Furthermore, reproducibility is an essential requirement of any screening test and has not yet been described before for the Cervista HPV HR test. In this study, we showed high intra-laboratory reproducibility and high inter-laboratory agreement, which even improved further by using the second threshold of 5.0.

The selection of our samples was based on the international guidelines for HPV DNA testing in primary cervical cancer screening in women 30 years and older by Meijer et al [24]. Nevertheless, the clinical sensitivity found in our dataset was comparable to literature. Literature shows that the sensitivity for the detection of CIN2+ is 85–100% for the HC2 test [2] and 90–100% for the Cervista HPV HR test [17], [21]. The corresponding clinical specificity is 84–96% for the HC2 test [2] and 68–91% for the Cervista HPV HR test [17], [21]. The sensitivity and specificity of the Cervista HPV HR test in a population-based setting was compared to the HC2 test in one large study (SHENCCASTII). In this population-based cross-sectional clinical study testing 8556 scrapings, the Cervista HPV HR test showed a sensitivity for CIN3+ of 95% and specificity of 90% similar as detected with the HC2 HPV test (98% and 88%, respectively) [21]. The HPV positivity rates in women with normal cytological results were 8% for HC2 and 6% for the Cervista HPV HR test in this cohort [21]. In two other studies using scrapings with a negative cervical cytology (NILM), no significant difference in prevalence rates was observed between the HC2 (5.9–7.5%) and Cervista HPV HR test (6.9–8.4%) [22], [23]. However, comparing data of the Cervista manufacturer's package insert [30] with data of different HC2 studies, Kinney et al. signaled that the Cervista HPV HR test was 2–4-fold more likely to give positive HPV test results in women >30 years with normal cytology compared to the HC2 test, suggesting that the Cervista HPV HR assay is significantly less specific than the HC2 assay [31]. Other studies do not reflect this opinion [16], [19]–[23]. Recently, Chateau et al. [19] compared a large data set generated from consecutive 9-month intervals of HC2 and Cervista HPV HR screening, stratified by age and cytological classification. Comparison of more than 1000 retrospective HC2 results from NILM patients aged >30 years to 1100 results generated by Cervista showed no difference in rates of detection. The authors describe that the overall Cervista detection rates in NILM patients (9.4%) in their study was similar to the detection rates from a meta-analysis of NILM patients (11.3%) [32]. These observations are in good agreement with the Cervista detection rate (10.0%) in our cohort of 900 women >30 years with normal cytology.

One of the limitations of the current FDA-approved HC2 test is the lack of an internal control. Without an internal control a negative HPV result could be due to the fact that the sample was hypocellular, the sample contained a substance that inhibited the signal amplification reaction or was processed incorrectly. The use of an internal control in the Cervista HPV HR test protects against a false-negative results due to these problems. In this study only 1 of the 965 scrapings gave a negative HC2-result whereas the internal control of the Cervista HPV HR test indicated that the sample had too few cells for reliable HPV-testing. Other studies comparing HC2 with the Cervista HPV HR test showed that the false-negative rate of the HC2 test due to insufficient input of cells is approximately 3.2–4.1% [16], [22]. An explanation for the low false-negative rate in our series is the fact that only samples with more than 12 ml PreservCyt solution were included to ensure that we would have sufficient material to compare both the HC2 and Cervista HPV HR assay, as well as to characterize discordant results. In general, residual samples with more than 12 ml contain higher cell counts since less PreservCyt is used to prepare cytological slides. The relatively low false-negative rate due to insufficient input of cells identified by the internal control has been suggested to be of limited benefit for the Cervista HPV HR test [22]. However, the potential of reducing the risk of false negatives by including the internal control in the Cervista HPV HR test becomes increasing important with primary HPV screening. The risk for women to develop CIN lesions will increase significant for HPV false-negative women, especially because in the suggested primary HPV screening program longer screening interval are advised [33].

In the cytological negative cases, 60 HC2-negative scrapings were positive by the Cervista HPV HR test. The GP5+/6+ PCR revealed only 3 HPV-positive cases suggesting a Cervista HPV HR false-positivity rate of 95% (57/60). Remarkably, of the 57 HPV-negative/Cervista-positive cases, 56% (32/57) were Cervista triple-positive defined as FOZ-ratio negative (<1.525) but considered HPV-positive because all three mixes had FOZ value higher than the second cut-off 1.93 (default setting). Comparison of the HC2-negative/Cervista triple-positive cases with the HC2-positive/Cervista-triple-positive cases revealed that changing the second cut-off to 5.0 improved the specificity significantly (Figure 1). While all five cases with CIN2/3 lesions were still positive for the Cervista HPV HR test, all 44 scrapings with normal cytomorphology became negative.

This new second cut-off of 5.0 for the Cervista HPV HR test was evaluated in an independent external cohort (SHENCCASTII) [21]. Using the default setting (second cut-off 1.93) 28 triple positive cases were considered as Cervista HPV positive, although most (n = 24) presented with normal histology. With the second cut-off of 5.0 all 4 CIN2+ remained Cervista HPV positive, whereas 22 of the 24 histological normal cases are now considered Cervista HPV negative. Thus 22/24 underwent unnecessary colposcopy and that could have been prevented by using the cut-off of 5.0. This remarkable improvement is in good agreement with our data using the Dutch population and warrants serious consideration to change the second cut-off.

Improving specificity is an important issue when it comes to primary population-based HPV screening. Since the prevalence of CIN2+ lesions in a population-based screening setting is relatively low, even small changes in clinical specificity of the hrHPV test will have enormous effects on the number of unnecessary referrals to the gynecologist and associated costs.

In our series of 900 cases, we observed 32 (3.6%) triple-positive cases with normal cytology (NILM). Literature shows no other studies using the Cervista HPV HR test that elaborates on Cervista triple-positive cases. In the reference-lab at the department of Pathology, Brugge Belgium that routinely uses the Cervista HPV HR test as a triage test in women with ASCUS, the prevalence of triple-positive cases (default setting at 1.93) for 2010 until 2011 was, 3.7% (73/1974 cases) (unpublished data). These data illustrate that triple-positive cases are described both in NILM and ASCUS at a rate of ∼3.6%.

Re-testing the scrapings of the 32 triple-positive cases (at cut-off 1.93) revealed again triple-positivity in 56% (18/32). The inter-laboratory agreement showed that 7 of the 22 triple-positive cases detected in lab 1 were also triple-positive in lab 2. This suggests that the positivity did not occur occasionally but is associated with the sample. The triple-positive result is partly due to the presence of various different HPV types in the sample. However, since in almost all cases with threshold <5.0 no HPV could be detected with HC2 and highly sensitive PCR-based consensus HPV tests, an HPV-unrelated factor might result in the increased FOZ value in all three mixes. In our series of 900 women with NILM scrapings no association was observed with age when comparing the triple-positive group (median age is 50 years; IQR 40.75–55) with the total group (median age is 46 years; IQR 40–55; p = 0.15). Although the use of vaginal anti-fungal creams or contraceptive jelly (not available from this study) did not seem to affect the positivity rate of the Cervista HPV HR test [13], a possible effect on Cervista outcome would also affect the HC2 result.

The reproducibility of the Cervista HPV HR test has not been described before. In this study we reported a high intra-laboratory reproducibility (92%; kappa 0.83) and high inter-laboratory agreement (90%; kappa 0.80). Using the second threshold of 5.0 the intra-laboratory reproducibility improved to 93% (kappa 0.84) and the inter-laboratory agreement to 93% (kappa 0.85).

In conclusion, the performance to detect hrHPV using the Cervista HPV HR test is comparable to the HC2 test regarding the sensitivity for detecting CIN2+ lesions. Data from this study in addition to external validation using the SHENCCASTII dataset demonstrate that increasing the second cut-off from default setting (1.93) to 5.0 will significantly improve the specificity of the Cervista HPV HR test.

## Supporting Information

Table S1 **Characterization of discordant cases using the analytical-sensitive GP5+/6+ PCR, HPV-typing with the INNO-LiPA and Cervista retesting.**
(DOC)

Table S2 **Summary of the 32 Cervista triple-positive cases using the analytical-sensitive GP5+/6+ PCR and HPV-typing with INNO-LiPA analysis.**
(DOC)

Table S3 **Summary of the 31 additional Cervista triple-positive cases using the analytical-sensitive GP5+/6+ HPV PCR and HPV-typing with INNO-LiPA analysis.**
(DOC)

Table S4 **SHENCCAST data of 28 triple positive cases with available histological results.**
(DOC)
