# Supplementary material for: Comparing the Cervista HPV HR Test and Hybrid Capture 2 Assay in a Dutch Screening Population: Improved Specificity of the Cervista HPV HR Test by Changing the Cut-Off
Source: PLoS One. 2014 Jul 22;9(7):e101930. doi: 10.1371/journal.pone.0101930 (PMC4106783; doi:10.1371/journal.pone.0101930)
Supplement: Table S1 — Characterization of discordant cases using the analytical-sensitive GP5+/6+ PCR, HPV-typing with the INNO-LiPA and Cervista retesting. (DOC) [file pone.0101930.s001.doc]

*Table S1: Characterization of discordant cases using the analytical-sensitive GP5+/6+ PCR, HPV-typing with the INNO-LiPA and Cervista retesting.*

| ***Nr*** | ***HC2 result*** | ***Cervista result*** | ***GP5+/6+ PCR*** | ***Cervista re-test*** | ***INNO Lipa*** |
| --- | --- | --- | --- | --- | --- |
| 1 | Negative | Low gDNA | Negative | Low gDNA | - |
| 2 | Positive | Negative | Negative | Negative | HPV 33 |
| 3 | Positive | Negative | Positive | Negative | HPV51 |
| 4 | Positive | Negative | Positive | - | - |
| 5 | Positive | Negative | Positive | - | HPV33, 69, 71 |
| 6 | Positive | Negative | Positive | Positive | HPV53, 54, 66 |
| 7 | Negative | Positive | Negative | - | Negative |
| 8 | Negative | Positive | Negative | Positive | Negative |
| 9 | Negative | Positive | Negative | Negative | Negative |
| 10 | Negative | Positive* | Negative | Positive | Negative |
| 11 | Negative | Positive* | Negative | Positive | Negative |
| 12 | Negative | Positive | Negative | Negative | - |
| 13 | Negative | Positive* | Negative | Positive* | Negative |
| 14 | Negative | Positive* | Negative | Positive | - |
| 15 | Negative | Positive* | Negative | Positive | Negative |
| 16 | Negative | Positive* | Negative | Positive | Negative |
| 17 | Negative | Positive* | Negative | Positive* | Negative |
| 18 | Negative | Positive* | Negative | Negative | Negative |
| 19 | Negative | Positive* | Negative | Negative | Negative |
| 20 | Negative | Positive | Negative | Negative | - |
| 21 | Negative | Positive* | Negative | Positive | - |
| 22 | Negative | Positive* | Negative | Negative | - |
| 23 | Negative | Positive* | Negative | Positive* | - |
| 24 | Negative | Positive* | Negative | Positive* | Negative |
| 25 | Negative | Positive* | Negative | Positive* | - |
| 26 | Negative | Positive* | Negative | Positive | - |
| 27 | Negative | Positive | Negative | Negative | - |
| 28 | Negative | Positive* | Negative | Positive* | - |
| 29 | Negative | Positive* | Negative | Negative | Negative |
| 30 | Negative | Positive* | Negative | Positive* | Negative |
| 31 | Negative | Positive* | Negative | Negative | Negative |
| 32 | Negative | Positive | Negative | Positive | Negative |
| 33 | Negative | Positive | Negative | Positive* | - |
| 34 | Negative | Positive | Positive | Negative | HPV39, 56 |
| 35 | Negative | Positive | Positive | Negative | HPV16 |
| 36 | Negative | Positive | Negative | Negative | HPV54 |
| 37 | Negative | Positive | Negative | Positive* | Negative |
| 38 | Negative | Positive* | Negative | Positive* | HPV X |
| 39 | Negative | Positive | Negative | Negative | - |
| 40 | Negative | Positive* | Negative | Positive* | Negative |
| 41 | Negative | Positive* | Negative | Negative | - |
| 42 | Negative | Positive | Negative | Negative | Negative |
| 43 | Negative | Positive | Negative | Negative | Negative |
| 44 | Negative | Positive* | Negative | Positive* | Negative |
| 45 | Negative | Positive | Negative | Negative | Negative |
| 46 | Negative | Positive* | Negative | Negative | Negative |
| 47 | Negative | Positive* | Negative | Positive | - |
| 48 | Negative | Positive | Negative | Positive* | - |
| 49 | Negative | Positive | Negative | Negative | Negative |
| 50 | Negative | Positive | Negative | Negative | - |
| 51 | Negative | Positive* | Negative | Positive* | Negative |
| 52 | Negative | Positive | Negative | Negative | Negative |
| 53 | Negative | Positive* | Negative | Negative | - |
| 54 | Negative | Positive | Negative | Positive | - |
| 55 | Negative | Positive | Negative | Positive | - |
| 56 | Negative | Positive | Negative | Negative | - |
| 57 | Negative | Positive | Negative | Negative | - |
| 58 | Negative | Positive | Negative | Negative | - |
| 59 | Negative | Positive* | Negative | Positive* | HPV X |
| 60 | Negative | Positive* | Negative | Positive* | - |
| 61 | Negative | Positive | Positive | Negative | HPV44, 56 |
| 62 | Negative | Positive* | Negative | Positive* | - |
| 63 | Negative | Positive | Negative | Negative | - |
| 64 | Negative | Positive* | Negative | Positive | Negative |
| 65 | Negative | Positive* | Negative | Positive* | Negative |
| 66 | Negative | Positive | Negative | Positive | Negative |

* The orange labelled Cervista positive cases are the triple positive cases (FOZ ratio < 1.525 and second cutoff of 1.93)
