# Supplementary material for: Comparing the Cervista HPV HR Test and Hybrid Capture 2 Assay in a Dutch Screening Population: Improved Specificity of the Cervista HPV HR Test by Changing the Cut-Off
Source: PLoS One. 2014 Jul 22;9(7):e101930. doi: 10.1371/journal.pone.0101930 (PMC4106783; doi:10.1371/journal.pone.0101930)
Supplement: Table S2 — Summary of the 32 Cervista triple-positive cases using the analytical-sensitive GP5+/6+ PCR and HPV-typing with INNO-LiPA analysis. (DOC) [file pone.0101930.s002.doc]

*Table S2: Summary of the 32 Cervista triple-positive cases using the analytical-sensitive GP5+/6+ PCR and HPV-typing with INNO-LiPA analysis. Histology was not available because of normal cytomorphology.*

| **Nr** | **Age** | **HC2 ratio** | **GP 5+/6+ PCR** | **Cervista HPV FOZ ratio** | **FOZ**  **Mix 1** | **FOZ**  **Mix 2** | **FOZ**  **Mix 3** |
| --- | --- | --- | --- | --- | --- | --- | --- |
| 1 | 55 | 0.14 | Negative | 1.30 | 3.14 | 2.42 | 2.45 |
| 2 | 55 | 0.31 | Negative | 1.27 | 2.67 | 2.11 | 2.42 |
| 3 | 50 | 0.15 | Negative | 1.04 | 4.78 | 4.60 | 4.63 |
| 4 | 41 | 0.19 | Negative | 1.22 | 4.04 | 3.39 | 4.14 |
| 5 | 39 | 0.14 | Negative | 1.34 | 4.11 | 3.07 | 3.83 |
| 6 | 55 | 0.18 | Negative | 1.40 | 3.75 | 2.69 | 3.18 |
| 7 | 50 | 0.33 | Negative | 1.30 | 4.47 | 3.44 | 3.67 |
| 8 | 55 | 0.21 | Negative | 1.34 | 2.82 | 2.11 | 2.23 |
| 9 | 55 | 0.34 | Negative | 1.12 | 2.16 | 1.97 | 2.21 |
| 10 | 50 | 0.29 | Negative | 1.25 | 4.43 | 3.56 | 3.70 |
| 11 | 40 | 0.14 | Negative | 1.08 | 1.98 | 1.96 | 2.12 |
| 12 | 55 | 0.18 | Negative | 1.10 | 6.58 | 6.83 | 6.22 |
| 13 | 55 | 0.22 | Negative | 1.39 | 3.33 | 2.86 | 3.99 |
| 14 | 38 | 0.14 | Negative | 1.16 | 2.97 | 2.72 | 3.15 |
| 15 | 45 | 0.17 | Negative | 1.42 | 3.68 | 2.59 | 3.28 |
| 16 | 53 | 0.28 | Negative | 1.37 | 2.77 | 2.02 | 2.27 |
| 17 | 50 | 0.21 | Negative | 1.18 | 2.77 | 2.40 | 2.84 |
| 18 | 55 | 0.16 | Negative | 1.05 | 4.10 | 3.91 | 4.11 |
| 19 | 42 | 0.18 | Negative | 1.21 | 2.98 | 2.47 | 2.62 |
| 20 | 57 | 0.23 | Negative | 1.52 | 3.81 | 2.50 | 3.31 |
| 21 | 49 | 0.44 | Negative | 1.28 | 2.95 | 2.33 | 2.98 |
| 22 | 40 | 0.48 | Negative | 1.52 | 3.21 | 2.11 | 2.39 |
| 23 | 60 | 0.31 | Negative | 1.47 | 3.25 | 2.21 | 2.37 |
| 24 | 38 | 0.36 | Negative | 1.28 | 2.49 | 1.95 | 2.24 |
| 25 | 40 | 0.30 | Negative | 1.20 | 2.69 | 2.24 | 2.42 |
| 26 | 49 | 0.41 | Negative | 1.45 | 4.01 | 2.76 | 3.24 |
| 27 | 41 | 0.45 | Negative | 1.30 | 2.65 | 2.04 | 2.17 |
| 28 | 60 | 0.22 | Negative | 1.14 | 3.64 | 3.19 | 3.59 |
| 29 | 59 | 0.25 | Negative | 1.31 | 3.41 | 2.59 | 2.67 |
| 30 | 60 | 0.47 | Negative | 1.22 | 2.90 | 2.44 | 2.38 |
| 31 | 60 | 0.18 | Negative | 1.16 | 2.30 | 1.99 | 2.00 |
| 32 | 60 | 0.20 | Negative | 1.40 | 4.14 | 2.99 | 2.96 |
