# Supplementary material for: Comparing the Cervista HPV HR Test and Hybrid Capture 2 Assay in a Dutch Screening Population: Improved Specificity of the Cervista HPV HR Test by Changing the Cut-Off
Source: PLoS One. 2014 Jul 22;9(7):e101930. doi: 10.1371/journal.pone.0101930 (PMC4106783; doi:10.1371/journal.pone.0101930)
Supplement: Table S3 — Summary of the 31 additional Cervista triple-positive cases using the analytical-sensitive GP5+/6+ HPV PCR and HPV-typing with INNO-LiPA analysis. (DOC) [file pone.0101930.s003.doc]

*Table S3: Summary of the 31 additional Cervista triple-positive cases using the analytical-sensitive GP5+/6+ HPV PCR and HPV-typing with INNO-LiPA analysis.*

| ***Nr*** | ***hc2 ratio*** | ***Cervista HPV FOZ ratio*** | ***FOZ***  ***mix 1*** | ***FOZ***  ***mix 2*** | ***FOZ***  ***mix 3*** | ***Histology*** |
| --- | --- | --- | --- | --- | --- | --- |
| 33 | 2585.12 | 1.18 | 9.42 | 8.01 | 9.30 | CIN3 |
| 34 | 717.37 | 1.18 | 7.59 | 6.69 | 7.87 | CIN3 |
| 35 | 0.28 | 1.29 | 3.39 | 2.88 | 2.63 | Unknown^1^ |
| 36 | 0.18 | 1.50 | 4.66 | 3.11 | 3.49 | Normal |
| 37 | 0.18 | 1.22 | 2.39 | 1.96 | 2.22 | Neg Cytology^2^ |
| 38 | 0.14 | 1.24 | 3.52 | 2.85 | 3.35 | Neg Cytology^2^ |
| 39 | 0.19 | 1.28 | 2.77 | 2.16 | 2.35 | Neg Cytology^2^ |
| 40 | 16.53 | 1.35 | 3.38 | 2.51 | 3.21 | Neg Cytology^2^ |
| 41 | 12.32 | 1.24 | 2.62 | 3.21 | 3.24 | Unknown^1^ |
| 42 | 1543.03 | 1.28 | 8.34 | 7.94 | 10.14 | CIN1 |
| 43 | 2772.47 | 1.23 | 10.06 | 8.18 | 9.34 | CIN3 |
| 44 | 0.12 | 1.33 | 3.08 | 2.40 | 3.18 | Unknown^1^ |
| 45 | 1409.52 | 1.42 | 8.88 | 7.96 | 6.28 | CIN2 |
| 46 | 0.19 | 1.43 | 3.78 | 2.64 | 3.10 | Unknown^1^ |
| 47 | 0.28 | 1.37 | 2.72 | 1.98 | 2.21 | Neg Cytology^2^ |
| 48 | 0.25 | 1.41 | 2.43 | 3.13 | 3.44 | Neg Cytology^2^ |
| 49 | 0.18 | 1.08 | 2.01 | 2.09 | 2.17 | Neg Cytology^2^ |
| 50 | 1269.43 | 1.25 | 6.58 | 6.03 | 7.60 | CIN2 |
| 51 | 216.15 | 1.20 | 7.20 | 7.09 | 6.01 | Unknown^1^ |
| 52 | 1868.84 | 1.14 | 9.21 | 8.06 | 8.31 | Unknown^1^ |
| 53 | 0.26 | 1.39 | 3.08 | 2.22 | 2.97 | Neg Cytology^2^ |
| 54 | 0.13 | 1.16 | 2.51 | 2.17 | 2.22 | Unknown^1^ |
| 55 | 0.27 | 1.39 | 2.87 | 2.09 | 2.07 | Neg Cytology^2^ |
| 56 | 0.24 | 1.39 | 3.42 | 2.53 | 2.45 | Neg Cytology^2^ |
| 57 | 0.19 | 1.36 | 2.72 | 2.21 | 2.00 | Neg Cytology^2^ |
| 58 | 0.13 | 1.22 | 2.53 | 2.07 | 2.09 | Neg Cytology^2^ |
| 59 | 0.16 | 1.26 | 2.86 | 2.27 | 2.67 | Unknown^1^ |
| 60 | 0.19 | 1.07 | 1.95 | 1.93 | 2.06 | Unknown^1^ |
| 61 | 0.14 | 1.35 | 2.77 | 2.05 | 2.35 | Unknown^1^ |
| 62 | 0.32 | 1.40 | 2.99 | 2.14 | 2.54 | Neg Cytology^2^ |
| 63 | 701.64 | 1.10 | 8.14 | 8.73 | 8.97 | Unknown^1^ |

^1^  Since all patients in this study were stored in a separate, anonymous database (according to the Dutch Law for Human Medical Research), specific histological data could not be retrieved anymore for some patients

^2^ Since women without cytomorphological abnormalities by Dutch law are not referred for colposcopy, histology is not available. Because these patients were twice negative by cytology, in this study they were presumed to be without CIN lesion and marked as negative cytology.
