# Supplementary material for: Comparing the Cervista HPV HR Test and Hybrid Capture 2 Assay in a Dutch Screening Population: Improved Specificity of the Cervista HPV HR Test by Changing the Cut-Off
Source: PLoS One. 2014 Jul 22;9(7):e101930. doi: 10.1371/journal.pone.0101930 (PMC4106783; doi:10.1371/journal.pone.0101930)
Supplement: Table S4 — SHENCCAST data of 28 triple positive cases with available histological results. (DOC) [file pone.0101930.s004.doc]

*Table S4: SHENCCAST data of 28 triple positive cases with available histological results.*

| **Age** | **hc2 ratio** | **Cytology** | **Histology** | **FOZ Mix1** | **FOZ Mix2** | **FOZ Mix3** | **FOZ**  **ratio** |
| --- | --- | --- | --- | --- | --- | --- | --- |
| 32 | 1896.99 | ASCUS | CIN2 | 9.56 | 8.56 | 8.89 | 1.12 |
| 51 | 1079.20 | Normal | CIN2 | 8.73 | 9.19 | 8.23 | 1.11 |
| 35 | 384.92 | HSIL | CIN3 | 9.32 | 8.94 | 9.68 | 1.08 |
| 52 | 887.70 | ASCUS | CIN3 | 9.46 | 9.12 | 8.74 | 1.08 |
| 38 | 2136.70 | Normal | CIN1 | 8.50 | 7.86 | 9.14 | 1.16 |
| 57 | 2176.99 | ASC-H | CIN1 | 7.99 | 6.99 | 8.49 | 1.22 |
| 39 | 80.71 | ASCUS | CIN1 | 1.95 | 1.98 | 2.59 | 1.33 |
| 54 | 9.84 | Normal | Normal | 3.65 | 3.13 | 3.07 | 1.19 |
| 34 | 5.31 | ASCUS | CIN1 | 3.37 | 2.99 | 3.26 | 1.13 |
| 33 | 0.20 | Normal | Normal | 3.61 | 2.69 | 3.13 | 1.34 |
| 52 | 0.33 | Normal | Normal | 2.79 | 2.02 | 2.44 | 1.38 |
| 43 | 0.16 | Normal | CIN1 | 2.37 | 2.64 | 2.81 | 1.19 |
| 54 | 0.19 | Normal | Normal | 2.20 | 2.04 | 2.12 | 1.07 |
| 52 | 0.19 | Normal | Normal | 2.87 | 2.19 | 2.36 | 1.31 |
| 35 | 0.15 | Normal | CIN1 | 2.32 | 1.94 | 2.83 | 1.46 |
| 39 | 0.21 | Normal | Normal | 2.95 | 2.25 | 2.19 | 1.35 |
| 42 | 0.18 | Normal | Normal | 2.45 | 2.20 | 2.66 | 1.21 |
| 39 | 0.12 | Normal | Normal | 2.36 | 2.02 | 2.12 | 1.17 |
| 52 | 0.20 | Normal | CIN1 | 2.48 | 2.15 | 2.42 | 1.15 |
| 37 | 0.27 | Normal | CIN0 | 3.65 | 3.08 | 3.75 | 1.22 |
| 35 | 0.13 | Normal | CIN1 | 2.48 | 1.94 | 2.39 | 1.28 |
| 52 | 0.23 | Normal | CIN1 | 3.33 | 2.42 | 3.10 | 1.38 |
| 44 | 0.16 | Normal | CIN0 | 3.00 | 2.10 | 2.9 | 1.43 |
| 43 | 0.17 | Normal | Normal | 2.12 | 1.95 | 2.18 | 1.12 |
| 37 | 0.52 | Normal | CIN1 | 2.88 | 3.27 | 3.16 | 1.14 |
| 39 | 0.21 | Normal | CIN1 | 2.33 | 2.00 | 2.31 | 1.16 |
| 42 | 0.17 | Normal | CIN0 | 2.40 | 2.58 | 2.26 | 1.14 |
| 42 | 0.22 | Normal | CIN1 | 3.16 | 2.15 | 2.44 | 1.47 |
